# Television viewing time as a risk factor for frailty and functional limitations in older adults: results from 2 European prospective cohorts

**DOI:** 10.1186/s12966-017-0511-1

**Published:** 2017-04-26

**Authors:** Esther García-Esquinas, Elena Andrade, David Martínez-Gómez, Francisco Félix Caballero, Esther López-García, Fernando Rodríguez-Artalejo

**Affiliations:** 1 Department of Preventive Medicine and Public Health, School of Medicine, Universidad Autónoma de Madrid/IdiPaz and CIBER of Epidemiology and Public Health (CIBERESP), Calle del Arzobispo Morcillo 4, 28029 Madrid, Spain; 2 Department of Physical Education, Sport and Human Movement, Faculty of Teacher Training and Education, Universidad Autónoma de Madrid, Madrid, Spain; 3 Department of Psychiatry, Universidad Autónoma de Madrid and CIBER of Mental Health (CIBERSAM), Madrid, Spain; 4 IMDEA-Food Institute. CEI UAM+CSIC, Madrid, Spain

**Keywords:** Frailty, Physical function, Sedentary behavior

## Abstract

**Background:**

Sedentariness is an important risk factor for poor health. The main objective of this work was to examine the prospective association between television viewing time and indicators of physical function, mobility, agility, and frailty.

**Methods:**

Data came from two independent cohorts of community-dwelling older adults: the Seniors-ENRICA (*n* = 2392, 3.5 year follow-up), and the ELSA (*n* = 3989, 3.9 year follow-up). At baseline, television viewing and other sedentary behaviors were ascertained using interviewer-administered questionnaires. In the Seniors-ENRICA cohort overall physical function at baseline and follow-up was assessed using the physical component summary (PCS) of the SF-12 Health Survey. Measures for incident mobility and agility limitations in both cohorts were based on standardized questions, and incident frailty was measured with the Fried criteria. Analyses were adjusted for the main confounders, including physical activity at baseline. Results across cohorts were pooled using a random effects model.

**Results:**

Lower (worse) scores in the PCS were observed among those in the highest (vs. the lowest) tertile of television viewing time (b-coefficient:-1.66; 95% confidence interval:-2.81,-0.52; p-trend = 0.01). Moreover, the pooled odds ratios (95% CIs) for mobility limitations for the second and third (vs. the lowest) tertile of television viewing were 1.00 (0.84, 1.20) and 1.17 (1.00, 1.38); p-trend = 0.12, respectively. The corresponding results for agility limitations were 1.18 (0.97, 1.44) and 1.25 (1.03, 1.51); p-trend = 0.02. Results for incident frailty were 1.10 (0.80, 1.51) and 1.47 (1.09, 1.97); p-trend = 0.03. No association between other types of sedentary behavior (time seated at the computer, while commuting, lying in the sun, listening to music/reading, internet use) and risk of functional limitations was found.

**Conclusions:**

Among older adults, longer television viewing time is prospectively associated with limitations in physical function independently of physical activity.

**Electronic supplementary material:**

The online version of this article (doi:10.1186/s12966-017-0511-1) contains supplementary material, which is available to authorized users.

## Background

Aging comes with a decline in most physiological systems culminating in limited physical capacity. According to the 2004 *Survey of Health, Ageing and Retirement in Europe*, around 43% of European men and 60% of European women aged ≥50 years reported at least one limitation in mobility and functioning. Further, about 9% of men and 12% of women reported ≥1 limitations in activities of daily living [1]. This presents a major challenge to public health, as functional impairments are an important predictor of disability [2–5], institutionalization [4], hospitalization [3, 6] and death [7]. Hence, identifying modifiable determinants of functional ability decline is critical.

Older people spend most of their awake time in sedentary activities, defined by a low energy expenditure (≤1.5 METs) while sitting or reclined [8]. Sedentary time has been associated with an increased risk of cardiovascular disease, type 2 diabetes, cancer, all-cause and cause-specific mortality [9]. Among older adults, a growing body of evidence associates sedentary behaviors with functional limitations [10–18]. However, most of this evidence is limited by cross-sectional designs [10–15]. Additionally, the few existing prospective studies are either based on patients with osteoarthritis, [17, 18] focus on physical performance, [16, 17] or lack a standardized definition of frailty [18]. These prospective findings link sedentary time (measured by accelerometry [17, 18] or defined as self-reported television (TV) viewing time [16]) to declines in gait speed and chair stand rates [17], incident frailty [18] and lower usual walking speed in older adults [16].

Since time spent watching TV is the main component of sedentary time among older adults, [17, 19, 20] this study assesses the prospective association between the amount of TV viewing time and a range of validated measures of physical function (i.e. overall functioning, limitations in mobility or agility, and frailty). We analyze data from 2 independent cohorts of community-dwelling older adults: the Study on Nutrition and Cardiovascular Risk Factors in Spain (Seniors-ENRICA), and the English Longitudinal Study of Aging (ELSA) cohorts. Additionally, as far as we are aware, this study is the first to examine the prospective association between other types of sedentary activities (time seated at the computer, while commuting, lying in the sun, listening to music and reading) and the risk of functional limitations.

## Methods

### Study population and design

#### Seniors-ENRICA cohort

Baseline data collection was conducted between 2008 and 2010 as part of a larger cross-sectional study named ENRICA, in which participants were selected by stratified cluster sampling of the non-institutionalized adult population of Spain. Information was collected in three stages: a phone interview, -designed to collect data on socio-demographic factors, lifestyle and morbidity-, plus two home visits. During the first home visit, nurses collected blood and urine samples. Information on functional limitations was obtained during the second home visit [21]. Participants aged ≥60 years (*N* = 3289) were then invited to participate in a prospective study called Seniors-ENRICA [22]. Those who accepted (*N* = 2614) were followed through 2012, when a second wave of data was collected. Ninety-five participants (3.6%) died during follow-up. Of the remaining 2519 participants, we excluded 18 for lacking complete data on sedentary time variables and an additional 19 who had missing information on potential confounders. Further, for analyses involving the physical component summary (PCS) of the 12-item Short-Form Health Survey (SF-12), we excluded 90 individuals who lacked information on this variable, either at baseline or at follow-up. Our final sample consisted of 2392 participants (subsample 1). Similarly, for analyses based on mobility limitations, agility limitations or frailty, we excluded individuals with no complete information on these items (*n* = 184, *n* = 117 and *n* = 555, respectively), as well as those who had mobility limitations (*n* = 734), agility limitations (*n* = 848), of were already frail (*n* = 40) at baseline. Thus, analyses were performed on 1564 (subsample 2), 1517 (subsample 3) and 1887 (subsample 4) participants, respectively. All participants provided written informed consent, and the Clinical Research Ethics Committee of ‘La Paz’ University Hospital in Madrid approved the study.

#### ELSA cohort

Established in 2002–2003, ELSA is a biennial longitudinal study representative of men and women aged ≥50 living in private households in England [23]. Participants are interviewed every 2 years and have a nurse visit every 4 years. Information on socio-demographic, psychological, cognitive and health factors is collected using computer-assisted interviews and self-completion questionnaires. ELSA is harmonized with ageing studies in other countries to facilitate international comparisons. For the current analyses we used information from 6118 participants aged ≥60 years who participated in wave 4 (2008–2009) and were followed through wave 6 (2012–2013). In both waves, information was collected using personal interviews, and measures of physical function and anthropometry were performed during nurse visits. From the initial sample, we excluded 169 individuals without complete data on sedentary time, 77 with implausible values on sedentary time (all of them above the 99 percentile of the distribution), and 53 with no information on potential confounders. For analyses examining mobility limitations, agility limitations, or frailty, we also excluded participants with no information on those items at baseline or at follow-up (*n* = 1074, *n* = 1074, and *n* = 1633, respectively), as well as those with mobility limitations (*n* = 1667), agility limitations (*n* = 1743), or frailty at baseline (197). Therefore, analyses were performed on 3078 (subsample 5), 3002 (subsample 6) and 3989 (subsample 7) participants, respectively. The National Research Ethics Service (MREC/01/2/91) provided the ethical approval for ELSA.

### Study variables

A description of the main variables included in the manuscript by study cohort can be found in Table 1.

**Table 1:** Description of the main variables included in the manuscript by study cohort

Variables	Cohort	Description	Categories
Sociodemographic variables, lifestyle, obesity and reported comorbidity			
Age, sex	ELSA	Self-reported	
	Seniors-ENRICA	Self-reported	
Educational level	ELSA	Self-reported	<High school High school Some college College or above Unknown
	Seniors-ENRICA	Self-reported	≤Primary Seconday University
Tobacco consumption	ELSA	Self-reported	Never Former Current
	Seniors-ENRICA	Self-reported	Never Former Current
Comorbidities (Cardiovascular disease, diabetes, chronic lung disease, osteomuscular disease).	ELSA	Self-reported	Presence/absence of each of the studied comorbidities
	Seniors-ENRICA	Self-reported	Presence/absence of each of the studied comorbidities
BMI	ELSA	Weight and height measured under standardized conditions.	<25 kg/m^2^ 25-29.9 kg/m^2^ ≥30 kg/m^2^
	Seniors-ENRICA	Weight and height measured under standardized conditions	<25 kg/m^2^ 25-29.9 kg/m^2^ ≥30 kg/m^2^
Diet quality	ELSA	Not available	
	Seniors-ENRICA	MEDAS index	Tertiles
Physical activity	ELSA	Self-reported Information on the frequency of vigorous, moderate and mild exercise was multiplied by the metabolic equivalent value for each activity.	Quintiles
	Seniors-ENRICA	Self-reported. Information on work and leisure time physical activity was obtained using the EPIC Physical Activity Questionnaire.	Inactive Moderately inactive Moderately active Active
Sedentary behaviors			
Television viewing time	ELSA	Self-reported a) n° hours/day spent watching TV from Monday to Friday b) n° hours/day spent watching TV over the weekend	Tertiles
	Seniors-ENRICA	Self-reported a) n° of hours/day spent watching TV	Tertiles
Other sedentary behaviors	ELSA	Self-reported use of the internet and/or email	Yes/No
	Seniors-ENRICA	Self-reported n° of hours/day a) sitting in front of a computer b) reading c) listening to music d) commuting e) sunbathing in summer f) sunbathing in winter.”	Tertiles
Functional limitations			
Overall physical functioning	ELSA	Not available	
	Seniors-ENRICA	Physical component summary of the SF-12	
Mobility limitations	ELSA	Self-reported. Affirmative answer to ≥1 of the following questions: a) Do you experience difficulty lifting/carrying weights over 10 pounds? b) Do you experience difficulty climbing one flight of stairs without resting? c) Do you experience any difficulty walking 1/4 mile unaided?	Yes/No
	Seniors-ENRICA	Self-reported. Affirmative answer to ≥1 of the following questions: a) Do you experience any difficulty in picking up/carrying a shopping bag? b) Do you experience any difficulty in climbing one flight of stairs? c) Do you experience any difficulty in walking several city blocks (a few hundred meters)?	Yes/No
Agility limitations	ELSA	Self-reported. Affirmative answer to the question “Do you experience any difficulty stooping/kneeling/crouching?”	Yes/No
	Seniors-ENRICA	Self-reported. Affirmative answer to the question “Do you experience any difficulty in bending/kneeling”	Yes/No
Frailty	ELSA	Individuals with ≥1 of the following criteria: a) Weakness: Cohort specific lowest quintile of grip strength adjusted for sex and BMI b) Exhaustion: Affirmative response to ≥1 of the statements a. I felt that everything I did was a big effort in the last week b. I could not get going in the last week c) Weight loss: Loss of ≥10% of body weight since wave 2 or current BMI <18.5 kg/m^2^ d) Low physical activity: lowest quintile of the distribution of physical activity e) Slow walking speed: Lowest quintile in the distribution of gait speed taking account of sex and height.	Yes/No
	Seniors-ENRICA	Individuals with ≥1 of the following criteria: a) Weakness: Cohort specific lowest quintile of grip strength adjusted for sex and BMI b) Exhaustion: Affirmative response to ≥1 of the statements a. I felt that everything I did was a big effort in the last week b. I could not get going in the last week c) Weight loss: Unintentional loss of ≥4.5 kg of body weight in the preceding year d) Low physical activity: Walking ≤2.5 h/week in men or ≤2 h/week in women. e) Slow walking speed: Lowest quintile in the distribution of gait speed taking account of sex and height.	Yes/No

### Sedentary behavior

In the Seniors-ENRICA study, information on sedentary behaviors was obtained using the Nurses’ Health Study questionnaire validated in Spain [24]. Specifically, participants were asked to recall the usual number of hours/day spent a) watching TV; b) sitting in front of a computer; c) reading; d) listening to music; e) commuting; f) sunbathing in summer; and g) sunbathing in winter. In the ELSA study, participants were asked to recall “How many hours of television do you watch on an ordinary day or evening, that is, Monday to Friday?” and “How many hours of television do you normally watch in total over the weekend, that is, Saturday and Sunday?” Average daily time spent watching TV was calculated as [(weekday TV time x 5) + (Weekend TV time)]/7. Additionally, participants were asked if they used a computer for internet or email.

In both cohorts TV viewing time was divided into sex-specific tertiles with the highest reflecting the highest level of sedentary behavior. The use of tertiles avoids the assumption that there is a linear relationship between sedentary time and the studied outcomes. More specifically, we have used sex-specific tertiles because of the different distribution of the studied sedentary behaviors across sexes, and to avoid creating exceedingly small subgroups.

### Functional limitations

#### Overall physical functioning

The PCS questionnaire was used in the Seniors-ENRICA cohort to assess overall function. The 4 items of the PCS evaluate four health dimensions: physical functioning, role-physical, bodily pain and general health. Subjects’ answers to any given item receive a numerical score which, after coding, is ranked on a scale of 0–100. The PCS score is then standardized to a national norm with a mean of 50 and a standard deviation (SD) of 10 to allow comparing the PCS for each study participant against the mean score in the Spanish population. A higher score indicates better physical function [25]. The SF-12 questionnaire was not available in the ELSA cohort.

#### Mobility limitation

In the Seniors-ENRICA study we considered someone reporting mobility limitation when they provided an affirmative answer to any of the following questions: 1)“Do you experience any difficulty in picking up/carrying a shopping bag?”, 2)“Do you experience any difficulty in climbing one flight of stairs?”, or 3)“Do you experience any difficulty in walking several city blocks (a few hundred meters)?” [5] In the ELSA study the questions we used to define mobility limitation were slightly different. An individual was considered to report mobility limitation if they answered affirmatively to at least one of the following: 1)“Do you experience difficulty lifting/carrying weights over 10 pounds?” 2)“Do you experience difficulty climbing one flight of stairs without resting?”, or 3)“Do you experience any difficulty walking 1/4 mile unaided?”

#### Agility limitation

This variable was assessed using the question “Do you experience any difficulty in bending/kneeling” in the Seniors-Enrica [26], and the question “Do you experience any difficulty stooping/kneeling/crouching?” in the ELSA study.

#### Frailty

According to the criteria proposed by Fried et al [27], individuals meeting ≥3 of the following criteria were considered frail: 1) Weakness: defined in both studies as the cohort-specific lowest quintile of grip strength adjusted for sex and body mass index (BMI); strength was measured with a hand held dynamometer, and the highest value in two (seniors-ENRICA) or three (ELSA) consecutive measures was used in the analyses; 2) Exhaustion: defined in both cohorts as an affirmative response to any of two statements taken from the Center for Epidemiologic Studies Depression Scale: “I felt that everything I did was a big effort in the last week” or “I could not get going in the last week” [28]; 3) Weight loss: defined in the Seniors-ENRICA study as an unintentional loss of ≥4.5 kg of body weight in the preceding year, and in the ELSA cohort as either loss of ≥10% of body weight since wave 2 or current BMI < 18.5 kg/m^2^; 4) Low physical activity: defined in the Seniors-ENRICA cohort as walking ≤2.5 h/week in men and ≤2 h/week in women. In the ELSA-study an overall measure of physical activity (PA) was derived by multiplying the frequency of vigorous, moderate and mild exercise by the metabolic equivalent (MET) value for each activity (6, 3 and 1.5, respectively). Low PA was defined as the lowest sex-specific quintile of the distribution of this overall measurement. Finally, the fifth criterion was slow walking speed, assessed in both cohorts by measuring the time taken to walk a distance of 8 feet at usual pace. The test was repeated and the mean of the two measurements calculated. Walking speeds in the lowest quintile of the distribution according to sex and height were defined as low gait speed.

### Other variables

For both cohorts, baseline data included information on age, sex, educational status, and self-reported tobacco consumption. Participants also reported whether they had previously suffered from any of the following diseases: cardiovascular disease (ischemic heart disease, stroke, or heart failure), diabetes, chronic lung disease (asthma or chronic bronchitis), or osteomuscular disease (osteoarthritis or arthritis).

Baseline weight and height were measured using standard methods. We calculated BMI as (weight in kg)/(height in m)^2^. Normal weight was defined as a BMI <25, overweight as a BMI between 25-29.9, and obesity as a BMI ≥30.

In the Seniors-ENRICA cohort, food consumption was assessed with a computerized validated diet history developed from that used in the EPIC-cohort study in Spain [29], and adherence to the Mediterranean diet was summarized using the Mediterranean Diet Adherence Score (MEDAS) index. [30] We used Spanish food composition tables to calculate energy intake [29]. Unfortunately the ELSA-study did not include a diet history.

For Seniors-ENRICA participants, PA information was gathered using the EPIC-cohort questionnaire [31] and summarized according to the Cambridge Physical Activity Index. [32] The Cambridge index includes four categories of the sum duration of walking, cycling, and sports (hours/week); this sum is then cross-tabulated with occupational PA categories to assign participants into one of four groups (inactive, moderately inactive, moderately active, and active). Alternatively, the average number of hours/week spent in vigorous PA was used. How we defined the overall measure of PA for ELSA study is described above (see Frailty section).

### Statistical analysis

The association between baseline TV viewing time (modeled as tertiles) and PCS at follow-up was assessed using linear regression. In addition, we used logistic regression to evaluate how TV viewing time was associated with incident agility or mobility limitations and with frailty. Two regression models were built in each case. Model 1 adjusted for age, sex, and education; model 2 further adjusted for BMI, tobacco consumption, PA (using either the Cambridge index or the average number of hours/week spent in vigorous PA in the seniors-ENRICA cohort; and an overall measurement of PA in the ELSA study), cardiovascular disease, diabetes, chronic lung disease, and osteomuscular disease. In the Seniors-ENRICA study, model 2 also controlled for total energy intake and the MEDAS index. Linear regression models with follow-up PCS scores as dependent variable further adjusted for baseline PCS scores. Finally, the association between TV viewing time and onset of each individual frailty criterion was evaluated using logistic regression models controlling for the same covariates as in model 2 above. All aforementioned potential confounders were time-constant variables measured at baseline

We performed a random effects meta-analysis to combine the effect sizes obtained in both cohorts. Between-cohort heterogeneity was tested with the Chi-square-based Q statistic and quantified with the use of the I^2^ statistic [33]. Linear trends were evaluated using the generalized least squares for trend estimations of summarized dose-response data.

Using likelihood ratio tests, we tested for the potential interaction between TV watching tertiles and indicator variables for the following subgroups: sex (men/women), BMI (<25/25-29.9/≥30), leisure time PA (inactive/active), and diabetes (no/yes). These analyses were adjusted for the same covariates as model 2 above.

## Results

Tables 2 and 3 show the distribution of TV viewing time according to baseline characteristics of study participants in the Seniors-ENRICA and ELSA cohorts, respectively. Results are shown for the different sub-samples evaluated. Overall, women, participants with lower educational level, higher BMI and lower MEDAS index, as well as smokers and those who were inactive or suffered from diabetes or osteomuscular disease, spent more time watching TV than their counterparts. Mean TV time at baseline was also higher among individuals who developed mobility limitations, agility limitations or frailty (data not shown).

**Table 2:** Baseline characteristics of four subsamples from the Seniors-ENRICA cohort across increasing tertiles of television (TV) viewing time (h/day)

		Subsample 1: Analyses on the PCS of the SF-12 (*N* = 2392)				Subsample 2: Analyses on mobility limitations (*N* = 1564)					Subsample 3: Analyses on agility limitations (*N* = 1517)					Subsample 4: Analyses on frailty (*N* = 1882)				
		≤2 h/day	2.1-3 h/day	>3 h/day	*p*		≤2 h/day	2.1-3 h/day	>3 h/day	*p*		≤2 h/day	2.1-3 h/day	>3 h/day	*p*		≤2 h/day	2.1-3 h/day	>3 h/day	*p*
	*n*	*%*	*%*	*%*		*n*	*%*	%	%		*n*	*%*	%	%		*n*	*%*	%	%	
Sociodemographic factors																				
Age																				
60-65	920	58.6	23.3	18.1		681	61.1	22.2	16.7		672	60.4	23.1	16.5		713	59.9	22.7	17.4	
66-71	774	52.7	22.6	24.7		513	56.7	22.2	21.1		492	54.9	24.0	21.1		595	53.6	22.9	23.5	
≥ 72	698	43.4	25.1	31.5	<0.01	370	45.4	24.6	30.0	<0.01	353	45.6	25.8	28.6	<0.01	574	43.4	26.0	30.6	<0.01
Sex																				
Men	1129	62.1	27.0	23.8		850	57.2	23.3	19.5		811	60.0	24.4	19.6		912	56.0	23.9	20.1	
Women	1263	62.9	29.4	34.0	<0.01	714	54.5	22.1	23.4	0.18	706	54.3	23.5	22.2	0.45	970	49.9	23.6	26.5	<0.01
Educational level																				
≤ Primary	1292	58.9	30.5	39.8		776	49.6	23.2	27.2		747	48.1	25.3	26.6		996	46.2	24.7	29.1	
Seconday	589	32.2	15.0	11.7		414	56.5	24.2	19.3		408	57.6	23.8	18.6		473	55.2	24.1	20.7	
University	511	33.9	10.9	57.8	<0.01	374	68.5	20.3	11.2	<0.01	362	67.1	21.6	11.3	<0.01	413	62.3	21.1	12.6	<0.01
Lifestyle																				
BMI (kg/m^2^)																				
< 25	464	64.0	19.8	16.2		349	67.1	18.1	14.8		357	64.2	19.6	16.2		369	65.1	20.3	14.6	
25-29.9	1179	52.4	25.7	21.9		795	55.1	24.9	20.0		795	55.1	25.5	19.4		942	52.9	25.6	21.5	
≥30	749	44.7	23.6	32.7	<0.01	420	48.3	22.6	29.1	<0.01	365	46.6	24.9	28.5	<0.01	571	45.0	22.9	32.1	<0.01
Smoking																				
Never	1404	53.4	22.6	24.0		863	57.9	21.6	20.5		835	57.6	23.0	19.4		1084	53.7	23.2	23.1	
Former	718	50.6	26.4	23.0		503	53.9	25.3	20.8		478	52.3	26.2	21.5		579	52.0	25.2	22.8	
Current	270	50.7	21.2	28.2	0.14	198	52.5	21.7	25.8	0.23	204	52.0	23.0	25.0	0.20	219	51.1	22.8	26.1	0.76
MEDAS (tertiles)^a^																				
≤ 6	799	49.6	22.0	28.4		500	56.4	20.0	23.6		500	54.4	23.4	22.2		642	51.4	21.5	27.1	
7-8	1023	52.3	23.6	24.1		657	55.3	22.7	22.1		631	54.2	23.6	22.2		763	52.5	24.3	23.2	
≥ 9	570	56.0	25.8	18.2	<0.01	407	56.5	26.3	17.2	0.07	386	57.8	25.4	16.8	0.29	447	55.7	26.0	18.3	0.02
Cambridge’ index																				
Inactive	1874	50.5	23.8	25.7		1196	54.4	23.0	22.6		1163	53.3	24.5	22.2		1472	51.2	23.7	25.1	
Active	518	58.7	23.0	18.3	<0.01	368	61.2	22.0	16.9	0.03	354	61.3	22.3	16.4	0.02	410	58.8	23.9	17.3	<0.01
Morbidity																				
Cardiovascular disease^b^																				
No	2268	52.0	23.8	24.2		1503	55.6	23.1	21.3		1457	55.0	24.2	20-8		1787	52.5	24.0	23.5	
Yes	124	57.2	29.4	23.4	0.44	61	63.9	14.8	21.3	0.29	60	58.3	20.0	21.7	0.76	95	60.0	20.0	20.0	0.36
Diabetes																				
No	2032	53.8	23.6	22.6		1351	57.3	23.2	19.5		1310	56.6	24.2	19.2		1603	54.3	23.7	22.0	
Yes	360	43.3	23.6	24.2	<0.01	213	47.0	20.2	32.8	<0.01	207	46.4	22.7	30.9	<0.01	2879	44.4	24.1	31.5	<0.01
Osteomuscular disease^c^																				
No	1218	55.9	24.6	19.5		920	60.0	21.9	18.1		917	58.2	23.5	18.3		991	56.8	24.1	19.1	
Yes	1174	48.5	22.5	29.0	<0.01	644	50.1	24.1	25.8	<0.01	600	50.5	24.8	24.7	<0.01	891	48.5	23.3	28.2	<0.01
Respiratory disease^d^																				
No	2217	52.5	23.7	23.8		1466	56.4	22.7	20.9		1415	55.6	24.8	20.6		1740	53.1	23.8	23.1	
Yes	175	49.7	21.7	28.6	0.36	98	49.0	24.5	26.5	0.30	102	50.0	25.5	24.5	0.51	142	50.0	23.2	26.8	0.60

ENRICA, Study on Nutrition and Cardiovascular Risk Factors in Spain

^a^Adherence to the Mediterranean diet (range 0-14); ^b^Self-reported ischemic heart disease, stroke, or heart failure; ^c^Self-reported hip or knee osteoarthritis or arthritis; ^d^Self-reported asthma or chronic bronchitis

**Table 3:** Baseline characteristics of three subsamples from the ELSA cohort across increasing tertiles of television (TV) viewing time (h/day)

	Subsample 5: Analyses on mobility limitations (*N* = 3078)					Subsample 6: Analyses on agility limitations (*N* = 3002)					Subsample 7: Analyses on frailty (*N* = 3989)				
		M: ≤3 h/d W: ≤3.6 h/d	M: 3-5 h/d W: 3.7-5.6 h/d	M: >5 h/d W: >5.6 h/d	*p*		M: ≤3 h/d W: ≤3.6 h/d	M: 3-5 h/d W: 3.7-5.6 h/d	M: >5 h/d W: >5.6 h/d	*p*		M: ≤3 h/d W: ≤3.6 h/d	M: 3-5 h/d W: 3.7-5.6 h/d	M: >5 h/d W: >5.6 h/d	*p*
	*n*	*%*	*%*	*%*		*n*	*%*	*%*	*%*		*n*	*%*	*%*	*%*	
Sociodemographic factors															
Age															
60-65	1362	43.6	30.6	25.8		1273	42.7	31.8	25.5		1600	38.5	30.9	30.6	
66-71	896	38.6	34.2	27.2		866	38.1	32.2	29.7		1133	31.7	32.0	36.3	
≥ 72	820	38.2	37.6	24.2	<0.01	863	38.0	36.0	26.0	0.03	1256	34.0	32.9	33.1	<0.01
Sex															
Men	1591	42.9	32.7	24.4		1519	41.9	32.6	25.5		1893	35.5	31.4	33.1	
Women	1487	38.3	34.4	27.3	0.03	1483	38.0	33.7	28.3	0.07	2096	34.8	32.2	33.0	0.32
Educational level															
< High school	788	24.8	35.7	39.5		854	25.2	35.1	29.7		1194	20.5	31.6	47.9	
High school	531	40.5	35.0	24.5		485	41.2	35.1	23.7		636	37.3	34.1	28.6	
Some college	567	47.3	33.3	19.4		516	45.1	35.8	19.1		692	42.5	33.2	24.3	
College or above	477	63.3	24.5	12.2		445	65.8	22.1	12.1		530	60.9	24.7	13.4	
Unknown	715	38.2	36.1	25.7	<0.01	702	37.0	34.5	28.5	<0.01	937	32.3	33.5	34.2	<0.01
Lifestlye															
BMI (kg/m^2^)															
< 25	770	50.0	29.2	20.8		821	47.8	29.1	23.1		1007	45.4	28.3	26.3	
25-29.9	1322	41.2	33.6	25.2		1257	40.9	33.6	25.5		1770	36.5	32.6	30.9	
≥ 30	986	32.8	36.7	30.5	<0.01	924	31.9	36.1	32.0	<0.01	1201	24.7	33.6	41.7	<0.01
Smoking															
Never	1255	42.8	33.6	23.6		1218	41.9	33.5	24.6		1580	39.2	31.8	29.0	
Former	1575	41.0	32.8	26.2		1494	40.7	32.2	27.1		2050	34.0	32.1	33.9	
Current	248	28.6	27.5	33.9	<0.01	290	28.6	36.6	34.8	<0.01	359	24.0	29.8	46.2	<0.01
Physical activity score															
< 10 METs-h/week	634	34.4	35.0	30.6		741	31.0	34.3	34.7		1349	27.5	32.7	39.8	
10-16 METs-h/week	1234	39.3	33.3	27.4		1149	39.7	34.3	26.0		1480	35.7	30.8	33.5	
> 16 METs-h/week	1210	45.5	32.9	21.6	<0.01	1112	46.3	31.1	22.6	<0.01	1160	43.3	32.1	24.6	<0.01
Morbidity															
Cardiovascular disease^a^															
No	2607	40.8	33.6	25.5		2482	40.2	33.2	26.6		3198	35.3	32.2	32.5	
Yes	471	40.1	32.7	27.2	0.76	520	39.0	32.7	28.3	0.73	791	34.6	30.2	35.2	0.33
Diabetes															
No	2856	41.4	33.4	25.2		2759	41.0	33.2	25.8		3635	36.3	31.9	31.8	
Yes	222	31.5	34.2	34.3	<0.01	243	28.8	32.1	39.1	<0.01	354	22.9	31.1	46.0	<0.01
Osteomuscular disease^b^															
No	2272	41.0	33.7	25.3		2216	40.2	32.7	27.1		2463	36.6	31.6	31.8	
Yes	851	38.8	32.0	29.2	0.29	786	39.4	34.4	26.2	0.68	1526	32.8	32.1	35.1	<0.01
Respiratory disease^c^															
No	2722	40.8	33.5	25.7		2610	40.3	33.3	26.4		3376	35.9	32.0	32.1	
Yes	356	40.5	33.5	26.0	0.99	392	37.8	32.1	30.1	0.29	613	31.2	30.7	37.1	0.01

ELSA, English Longitudinal Study of Ageing

^a^Self-reported ischemic heart disease or stroke; ^b^Self-reported arthritis: ^c^Self-reported lung disease

In the Seniors-ENRICA cohort, mean (SD) baseline and follow-up PCS scores were 45.5 (11.7) and 44.5 (12.4), respectively. Also, 30.0% of participants developed mobility limitations, 44.8% developed agility limitations, and 7.3% developed frailty over a mean (SD) follow-up period of 3.3 (0.6) years. Corresponding figures for the ELSA cohort were 47.1%, 48.4%, and 5.1% over a mean (SD) follow-up of 3.9 (0.2) years.

In Table 4, we present results regarding TV time and limitations in physical function. As results from basically-adjusted models (sociodemographic variables only), and fully- adjusted models were similar, we emphasize fully-adjusted results throughout. Compared to individuals in the lowest tertile of TV time, those in the highest tertile showed lower PCS scores (b-coefficient:-1.66; 95%CI:-2.81,-0.52); p-trend = 0.01). Further adjustment for the mental component summary of the SF-12 (b-coefficient: -1.81; (95%CI:-2.94,-0.67); p-trend < 0.01), yielded comparable results.

**Table 4:** Association between television viewing time and incident physical function limitations among community-dwelling older adults from two independent cohorts

		PCS			Mobility limitations			Agility limitations			Frailty		
Study cohort	Tertiles of TV viewing time (h/d)	Mean (SD)	Model 1 Beta (95% CI)	Model 2 Beta (95% CI)	n events/total	Model 1 OR (95% CI)	Model 2 OR (95% CI)	n events/total	Model 1 OR (95% CI)	Model 2 OR (95% CI)	n events/total	Model 1 OR (95% CI)	Model 2 OR (95% CI)
Seniors-ENRICA													
	T1: ≤2 (M and W)	45.8 (12.0)	-	-	236/875	1.00	1.00	341/837	1.00	1.00	49/996	1.00	1.00
	T2: 2.1-3 (M and W)	45.1 (11.6)	0.37 (-0.63,1.36)	0.04 (-1.08,1.15)	103/356	1.05 (0.79,1.40)	1.00 (0.74,1.34)	163/364	1.12 (0.87,1.45)	1.05 (0.81,1.37)	31/448	1.14 (0.70,1.85)	1.20 (0.73,1.97)
	T3: >3 (M and W)	41.2 (13.4)	-1.42 (-2.44,-0.41)	-1.66 (-2.81,-0.52)	131/333	1.47 (1.10,1.95)	1.25 (0.93,1.69)	175/316	1.58 (1.21,2.97)	1.40 (1.05,1.86)	57/443	1.91 (1.25,2.92)	1.60 (1.02,2.49)
	p-trend		0.02	0.01		0.01	0.19		<0.01	0.03		<0.01	0.04
	Per 1 h increase		-0.34 (-0.61,-0.08)	-0.41 (-0.70,-0.11)		1.10 (1.02,1.18)	1.05 (0.97,1.14)		1.09 (1.02,1.18)	1.06 (0.98,1.14)		1.17 (1.05-1.29)	1.10 (1.00-1.23)
ELSA													
	T1: ≤3 (M) and ≤3.6 (W)	-	-	-	226/1253	1.00	1.00	233/1201	1.00	1.00	55/1402	1.00	1.00
	T2: 3-5 (M) and 3.7-5.6 (W)	-	-	-	221/1031	1.13 (0.91,1.41)	1.00 (0.80,1.26)	264/995	1.40 (1.13,1.73)	1.29 (1.03,1.60)	60/1269	1.15 (0.77,1.70)	1.03 (0.68,1.55)
	T3: >5 (M) and >5.6 (W)	-	-	-	186/794	1.38 (1.10,1.74)	1.14 (0.89,1.45)	210/807	1.36 (1.09,1.71)	1.15 (0.91,1.47)	88/1318	1.74 (1.19,2.53)	1.37 (0.93,2.04)
	p-trend					<0.01	0.32		<0.01	0.19		<0.01	0.09
	Per 1 h increase					1.03 (1.00,1.05)	1.01 (0.99,1.04)		1.03 (1.00,1.05)	1.02 (0.99,1.03)		1.02 (0.98,1.06)	1.00 (0.96,1.05)
Random effects meta-analysis													
	T1				462/2128	1.00	1.00	574/2038	1.00	1.00	104/2398	1.00	1.00
	T2				324/1387	1.10 (0.92,1.31)	1.00 (0.84,1.20)	427/1359	1.27^a^(1.02,1.57)	1.18 (0.97,1.44)	91/1717	1.15 (0.84,1.56)	1.10 (0.80,1.51)
	T3				317/1127	1.41 (1.18,1.69)	1.17 (1.00,1.38)	385/1123	1.40 (1.15,1.71)	1.25 (1.03,1.51)	145/1761	1.81 (1.37,2.40)	1.47 (1.09-1.97)
	p-trend					<0.01	0.12		<0.01	0.02		<0.01	0.03
	Per 1 h increase					1.05 (1.00,1.12)	1.01 (0.99,1.04)		1.05 (1.00,1.10)	1.02 (1.00,1.04)		1.08 (0.95,1.24)	1.05 (0.95,1.13)

ENRICA, Study on Nutrition and Cardiovascular Risk Factors in Spain; ELSA: English Longitudinal Study of Ageing

T: Tertile (T1, T2 and T3: Tertiles 1, 2 and 3)

M: Men; W:Women. OR: Odds ratio; CI: Confidence interval

Model 1 was adjusted for age, sex and educational level

Model 2 was adjusted for age, sex, educational level, BMI (<25, 25-29.9, ≥30 kg/m2), tobacco (never, former, current smoker), Mediterranean Diet Adherence Scale (MEDAS) index, total energy intake (kcal/day), physical activity, cardiovascular disease, diabetes mellitus, respiratory disease and osteo-muscular disease. All linear regression models were also adjusted for the baseline PCS score. Note that in the ELSA study no adjustment was made for the MEDAS index or for energy intake

^a^I^2^ > 30%; data should be interpreted with caution. Beta coefficients and their 95% confidence intervals were obtained from multiple lineal regression models. Odds ratios and their 95% confidence intervals were obtained from multiple logistic regression models

Results from the two cohorts have been pooled using random-effects meta-analysis

The pooled odds ratios [ORs] (95%CI) for mobility limitations comparing the second and third to the lowest tertile of TV viewing were1.00 (0.84, 1.20) and 1.17 (1.00, 1.38), respectively. Corresponding ORs for agility limitations were 1.18 (0.97, 1.44) and 1.25 (1.03, 1.51); and 1.10 (0.80, 1.51) and 1.47 (1.09, 1.97) for incident frailty. Effect modification by sex, BMI, diabetes, or PA level was not observed in any of the cohorts (see Additional files 1 and 2: Tables S1 and S2).

Results for the association between TV time and each individual frailty criterion are shown in Table 5. The OR (95%CI) from pooled analyses showed a non-statistically significant increased risk of exhaustion (1.16 (0.98, 1.38)) and low PA (1.17 (0.90, 1.52)) among individuals in the third tertile of TV time. Further, we observed an increased risk of weakness (*p* = 0.02) as time spent watching TV lengthened.

**Table 5:** Association between television viewing time at baseline and risk of each frailty criterion in two independent cohorts of community-dwelling older adults

		Exhaustion		Low physical activity		Slow walking speed		Weakness		Weight loss	
Study cohort	Tertiles of TV viewing time (h/d)	n events/total	OR (95% CI)	n events/total	OR (95% CI)	n events/total	OR (95% CI)	n events/total	OR (95% CI)	n events/total	OR (95% CI)
Seniors-ENRICA											
	T1: ≤2 (M and W)	115/996	1.00	147/996	1.00	136/984	1.00	284/993	1.00	78/996	1.00
	T2: 2.1-3 (M and W)	50/448	0.85 (0.59,1.23)	64/448	0.89 (0.64,1.23)	68/442	1.06 (0.77,1.46)	166/447	1.30 (1.00,1.69)	32/409	0.78 (0.50,1.21)
	T3: >3 (M and W)	88/443	1.22 (0.87,1.69)	84/443	1.00 (0.73,1.37)	72/435	0.99 (0.71,1.38)	202/443	1.33 (1.02,1.74)	38/398	0.74 (0.49,1.14)
	p-trend		0.32		0.92		0.99		0.02		0.15
	Per 1 h increase		1.05 (0.97,1.14)		1.00 (0.92,1.08)		1.00 (0.92,1.08)		1.08 (1.00,1.16)		0.94 (0.84,1.05)
ELSA											
	T1: ≤3 (M) or ≤3.6 (W)	269/1397	1.00	224/1402	1.00	346/1297	1.00	274/1377	1.00	59/1402	1.00
	T2: 3-5 (M) or 3.7-5.6 (W)	279/1266	0.97 (0.80,1.19)	260/1269	1.09 (0.87,1.36)	215/1176	0.77 (0.63,0.95)	279/1238	1.07 (0.86,1.36)	73/1269	1.27 (0.88,1.83)
	T3: >5 (M) or >5.6 (W)	365/1315	1.14 (0.93,1.40)	353/1318	1.31 (1.05,1.63)	183/1204	0.74 (0.60,0.92)	306/1290	1.13 (0.89,1.43)	84/1318	1.31 (0.90,1.89)
	p-trend		0.18		0.02		<0.01		0.31		0.17
	Per 1 h increase		1.00 (0.98,1.03)		1.01 (0.99,1.04)		0.97 (0.94,0.99)		1.00 (0.98,1.03)		1.00 (0.96,1.04)
Random effects meta-analysis											
	T1	384/2393	1.00	371/2398	1.00	482/2281	1.00	558/2370	1.00	137/2398	1.00
	T2	329/1714	0.94 (0.79,1.12)	324/1717	1.02 (0.85,1.23)	283/1618	0.88^a^(0.65,1.20)	445/1685	1.17 (0.97,1.41)	105/1678	1.01^a^ (0.63,1.63)
	T3	453/1758	1.16 (0.98,1.38)	437/1761	1.17^a^ (0.90,1.52)	255/1639	0.83^a^(0.63,1.10)	508/1733	1.21 (1.03,1.45)	122/1716	0.99^a^ (0.57,1.74)
	p-trend		0.47		0.56		0.19		0.02		0.99
	Per 1 h increase										

ENRICA, Study on Nutrition and Cardiovascular Risk Factors in Spain; ELSA: English Longitudinal Study of Ageing

T: Tertile (T1, T2 and T3: Tertiles 1, 2 and 3) M: Men; W: Women. OR: Odds ratio; CI: Confidence interval

Odds ratios and their 95% confidence intervals were obtained from multiple logistic regression models

Models were adjusted for age, sex, educational level, body mass index (<25, 25-29.9, ≥30 kg/m2), tobacco (never-, former current-smoker), total energy intake (kcal/day), Mediterranean Diet Adherence Scale (MEDAS) index, physical activity, cancer, diabetes, cardiovascular disease, osteomuscular disease and chronic respiratory disease. Note that in the ELSA study no adjustment was made for the MEDAS index or for energy intake

^a^I^2^ > 30%; data should be interpreted with caution

Results from the two cohorts have been pooled using random-effects meta-analysis

As ancillary analyses, we examined associations between five types of sedentary activities (other than watching TV) such as time seated at the computer, while commuting, lying in the sun, listening to music, and reading, and the risk of functional limitations (Seniors-ENRICA study); and the association between internet usage (no/yes) and the risk of functional limitations (ELSA cohort). Fully-adjusted analyses yielded no associations between most of these activities and physical function (Table 6). However, computer use seemed to have certain beneficial effect in both cohorts. Time seated at the computer showed a trend toward more favorable SF-12 scores (*p* = 0.05), and internet usage was associated with a decreased risk of agility limitations (OR: 0.76; 95% CI:0.62,0.93) and frailty (OR:0.64; 95% CI:0.43,0.95).

**Table 6:** Association between tertiles of time in sedentary behaviors other than TV viewing and incident limitations in physical function among community-dwelling older adults from two independent cohorts

Study cohort	Sedentary behavior, time tertiles (h/d)	Physical Health Composite Score			Mobility limitations		Agility limitations		Frailty	
		n	Mean (SD)	Beta (95% CI)	n events/total	OR (95% CI)	n events/total	OR (95% CI)	n events/total	OR (95% CI)
	Computer/Internet use									
Seniors-ENRICA										
	T1: 0 (M and W)	1676	43.1 (12.9)		365/1052	1.00	508/1036	1.00	125/1337	1.00
	T2: 0.1-0.4 (M) or 0.1-0.6 (W)	250	48.3 (10.1)	1.36 (-0.20,2.91)	42/183	0.88 (0.58,1.34)	70/176	0.99 (0.69,1.42)	2/203	0.28 (0.07,1.18)
	T3: >0.4 (M) or >0.6 (W)	437	48.0 (10.4)	1.20 (-0.14,2.55)	63/329	0.81 (0.57,1.16)	305/101	0.74 (0.54,1.02)	10/342	0.81 (0.38,1.71)
	p-trend			0.05		0.24		0.08		0.33
ELSA										
	No use (=0 h/day)		-	-		1.00		1.00		1.00
	Use (>0.1 h/day)		-	-		0.82 (0.67,1.01)		0.76 (0.62,0.93)		0.64 (0.43,0.95)
	Reading									
Seniors-ENRICA										
	T1: <0.3 (M) or <0.1 (W)	898	42.8 (13.1)		183/553	1.00	248/513	1.00	67/667	1.00
	T2: 0.3-1.0 (M) or 0.2-1.0 (W)	969	45.3 (11.9)	1.15 (0.09,2.22)	187/649	0.79 (0.60,1.05)	270/610	0.95 (0.73,1.23)	45/772	0.81 (0.52,1.25)
	T3: >1.0 (M and W)	615	45.9 (11.9)	1.09 (-0.15,2.33)	100/382	0.86 (0.62,1.21)	161/394	0.89 (0.66,1.19)	25/443	0.83 (0.48,1.44)
	p-trend			0.07		0.31				0.42
	Listening to music									
Seniors-ENRICA										
	No (=0 h/day)	1971	44.4 (12.5)	1.00	364/1213	1.00	523/1177	1.00	117/1482	1.00
	Yes (>0.1 h/day)	511	44.9 (12.3)	-0.47 (-1.58,0.64)	106/351	1.18 (0.89,1.57)	156/340	1.18 (0.91,1.54)	20/400	0.72 (0.42,1.22)
	Transportation									
Seniors-ENRICA										
	T1: 0 (M and W)	1208	43.2 (12.8)	1.00	266/736	1.00	349/699	1.00	86/934	1.00
	T2: 0.1-0.4 (M) or 0.1-0.2 (W)	609	45.8 (11.7)	0.30 (-0.85,1.44)	88/422	0.72 (0.53,0.99)	151/418	0.71 (0.54,0.93)	29/480	1.26 (0.76,2.08)
	T3: >0.4 (M) or >0.2 (W)	575	45.9 (12.1)	0.60 (-0.54,1.74)	116/406	0.91 (0.67,1.21)	179/400	0.94 (0.72,1.23)	22/468	0.91 (0.54,1.55)
	p-trend			0.30		0.34		0.47		0.94
	Sunbathing									
Seniors-ENRICA										
	No (=0 h/day)	1841	44.1 (12.7)	1.00	355/1172	1.00	517/1126	1.00	119/1442	1.00
	Yes (>0.1 h/day)	551	46.1 (11.4)	0.87 (-0.21,1.95)	392/115	0.93 (0.71,1.23)	162/391	0.85 (0.66,1.09)	18/440	0.63 (0.37,1.01)

ENRICA, Study on Nutrition and Cardiovascular Risk Factors in Spain; ELSA: English Longitudinal Study of Ageing

T: Tertile (T1, T2 and T3: Tertiles 1, 2 and 3); M: Men; W: Women. OR: Odds ratio; CI: Confidence interval

Beta coefficients and their 95% confidence intervals were obtained from multiple lineal regression models. Odds ratios and their 95% confidence intervals were obtained from multiple logistic regression models

Models were adjusted for age, sex, educational level, body mass index (<25, 25-29.9, ≥30 kg/m2), tobacco (never-, ex-, current-smoker), total energy intake (kcal/day), MEDAS index, physical activity, cancer, diabetes, cardiovascular disease, osteomuscular disease and chronic respiratory disease. All linear regression models were also adjusted for the baseline PCS score. Note that in the ELSA study no adjustment was made for the MEDAS indexor for energy intake

Results from the two cohorts have been pooled using random-effects meta-analysis

## Discussion

Our results show an association between time spent watching TV and an increased risk for unfavorable outcomes in physical functioning. These associations persisted after accounting for a range of covariates, including physical activity.

Cross-sectional studies have recently linked time spent watching TV with lower (worse) PCS scores (SF-36) [12], lower mean grip strength, [11, 14] lower timed Up-&-Go scores [11], and higher prevalence of IADL [10] and ADL [13] limitations in older adults. Further, evidence from newly-published longitudinal research deems sedentary time as a likely risk factor for functional decline [16–18]. Of these studies, two are based on the Osteoarthritis Initiative database, and connect accelerometer-based total sedentary time with declines in gait speed and chair stand rates [17], as well as with incident frailty, defined as low gait speed (<0.6 m/second) or inability to perform a single chair stand [18]. The third study, using data from 8,623 community-based participants enrolled in the EPIC-Norfolk, ascertained that watching TV for longer times is associated with lower usual walking speed, but not with lower grip strength [16].

Our results support the existence of a direct association between sedentary time and physical weakness, while contributing evidence indicating that time spent watching TV may be an important risk factor of agility limitations and frailty. Our results also suggest that health policy interventions should target heavy television viewers. In this subpopulation, just relatively small reductions in time in front of the TV set (i.e., moving from the third to the second tertile of TV viewing) could substantially reduce the risk of suffering physical limitations. Whereas the adverse consequences of excessive time spent watching TV could be palliated by increasing moderate or vigorous physical activity [34],the fact that our associations were independent of physical activity level suggests that harmful effects may also be reduced with no substantial modification of total activity For instance, by replacing time spent seated or reclined watching TV with time in a standing position browsing the computer screen. Future research should assess the feasibility and effectiveness of such interventions.

In contrast to our TV time-related findings, we failed to discern additional associations between other sedentary activities and functional limitations. Findings from previous cross-sectional research in older adults discriminated between associations of *passive* sedentary time (TV time, listening or talking while sitting, and sitting around) and *mentally-active* sedentary time (consisting of computer-use and reading books or newspapers) with health-related attributes, such as obesity and moderate-vigorous activity [20]. Similarly, cross-sectional findings from the ELSA cohort (wave 4) indicate that whereas internet usage was associated with stronger grip strength, time spent watching TV was linked to weaker strength, supporting our TV time-related results. The reasons behind these contrasting associations are not known. A potential explanation is that watching TV entails specific health risks beyond those expected from being seated [35]. Also, one could speculate that the amount of time spent watching TV is more easily recalled than other sedentary activities, that the time spent in these behaviors is relatively small (making it difficult to assess their full impact on health), or that these behaviors differ from TV watching in their association with potential confounders; thus adjusting for the same set the covariates may lead to different residual confounding.

TV watching could influence the risk of functional limitations through several mechanisms. First, longer periods of time spent sitting have been associated with a greater risk of sarcopenia [36], a major cause of functional limitations in the elderly [37, 38]. Actually, time spent sitting or lying down is the only state characterized by absence of muscle contraction, which may affect muscle metabolism independently of total PA. In fact, experimentally reducing normal spontaneous standing and ambulatory time had a much greater effect on the regulation of skeletal muscle lipoprotein lipase (important for controlling plasma triglyceride catabolism, HDL cholesterol, and other metabolic risk factors) than adding vigorous exercise training on top of normal non-exercise activity [39]. Second, sedentary behavior has been related to a higher risk of several pathologic conditions (e.g., cardiovascular disease), themselves important risk factors for functional limitations [40]. Finally, there is some evidence that sedentariness increases inflammation [41] which, in turn, may play a role in the development of functional limitations [42].

Cross-sectional studies focusing on characteristics of sedentary behavior other than its duration showed that daily breaks in sedentary time are associated with better leg function [43], improved lower limb extensor muscle quality [44], higher scores in the Senior Fitness Test [45], and lower risk for ADL impairments [45]. In this context, several intervention studies evaluating the feasibility of increasing the number of breaks in prolonged sedentary time are being conducted, with encouraging results [46, 47]. Recently, a published meta-analysis of randomized controlled trials also showed the effectiveness of step-counter use in walking programs to reduce sedentary time among older adults [48]. Future research should assess whether appropriate interventions addressing sedentariness can reduce the risk of functional limitations.

Our analysis has several strengths. First, the Seniors-ENRICA and ELSA cohorts had a prospective design, which allows for the appropriate time sequence between sedentary time and functional limitations. Second, in both cohorts physical function was ascertained with validated measures, including a standardized definition of frailty according to the Fried criteria, and physical performance tests were conducted by trained staff under standardized conditions. Finally, we considered a wide variety of function impairments, from less severe problems such as mobility or agility limitations, to more severe, such as frailty.

The main limitation of the study was its reliance on self-reported information. And, we could not evaluate the presence of breaks in sedentary time, which, as mentioned above, may be an important factor in the associations examined. Also, we could not evaluate the association between sedentary time and PCS score in the ELSA cohort since the SF-12 questionnaire was not available. Finally, although we adjusted our results for a large number of potential confounders, certain residual confounding cannot be ruled out because TV watching is strongly associated with the presence of unhealthy behaviors (i.e. unhealthy diet) [49], and with lower socioeconomic status [50], factors that have been associated with impaired physical function.

## Conclusions

Our study suggests that time spent watching TV is associated with an increased risk of several functional limitations in older adults. Thus, our study adds to current knowledge on sedentary behavior and its harmful effects by focusing on outcomes other than diseases, and suggests that replacing TV time by time spent standing or in light or more intense physical activity, according to the abilities of each individual, could delay physical impairment in the old age. Notwithstanding this, prospective studies including objective measures of sedentary behavior and characterization of breaks in sedentary time should further evaluate the relationship between sedentary behavior and physical limitations.

## Additional files

Additional file 1: Table S1. Stratified results for the association between tertiles of TV viewing time and incident limitations in physical function in older adults from the Seniors-ENRICA cohort. (DOCX 17 kb)

Additional file 2: Table S2. Stratified results for the association between tertiles of TV viewing time and incident limitations in physical function in older adults from the ELSA cohort. (DOCX 17 kb)
