# Supplementary material for: Television viewing time as a risk factor for frailty and functional limitations in older adults: results from 2 European prospective cohorts
Source: Int J Behav Nutr Phys Act. 2017 Apr 26;14:54. doi: 10.1186/s12966-017-0511-1 (PMC5406978; doi:10.1186/s12966-017-0511-1)
Supplement: Supplementary file 1 — Stratified results for the association between tertiles of TV viewing time and incident limitations in physical function in older adults from the Seniors-ENRICA cohort. (DOCX 17 kb) [file 12966_2017_511_MOESM1_ESM.docx]

**Additional file 1: Table S1: Stratified results for the association between tertiles of TV viewing time and incident limitations in physical function in older adults from the Seniors-ENRICA cohort .**

|  | | **Physical Health**  **Composite Score**  Beta (95% CI) | | | |  | **Mobility limitations**  OR (95% CI) | | | |  | **Agility limitations**  OR (95% CI) | | | |  | **Frailty**  OR (95% CI) | | | |
| --- | --- | --- | --- | --- | --- | --- | --- | --- | --- | --- | --- | --- | --- | --- | --- | --- | --- | --- | --- | --- |
|  | **Hours/day** | | | |  |  | **Hours/day** | | |  |  | **Hours/day** | | |  |  | **Hours/day** | | |  |
|  | **≤2** | | **2.1-3** | **>3** |  |  | **≤2** | **2.1-3** | **>3** |  |  | **≤2** | **2.1-3** | **>3** |  |  | **≤2** | **2.1-3** | **>3** |  |
|  |  | |  |  | **p*** |  |  |  |  | **p*** |  |  |  |  | **p*** |  |  |  |  | **p*** |
| **Sex** |  | |  |  |  |  |  |  |  |  |  |  |  |  |  |  |  |  |  |  |
| Men | Ref | | 0.17  (-1.42;1.76) | -2.17  (-3.87;-0.48) |  |  | Ref | 0.99 (0.64;1.55) | 1.36 (0.87;2.12) |  |  | Ref | 1.24 (0.86;1.77) | 1.45 (0.98;2.15) |  |  | Ref | 0.64 (0.24;1.68) | 1.43 (0.66;3.07) |  |
| Women | Ref | | -0.08  (-1.62;1.46) | -1.28  (-2.79;0.22) | 0.65 |  | Ref | 1.00 (0.67;1.49) | 1.17 (0.79;1.74) | 0.87 |  | Ref | 0.87 (0.59;1.28) | 1.34 (0.89;2.01) | 0.42 |  | Ref | 1.57 (0.86;2.85) | 1.66 (0.97;2.84) | 0.27 |
| **BMI(kg/m^2^)** | | |  |  |  |  |  |  |  |  |  |  |  |  |  |  |  |  |  |  |
| <25 | Ref | | -0.34  (-2.94;2.26) | -1.17  (-4.01;1.67) |  |  | Ref | 0.69 (0.35;1.39) | 1.36 (0.68;2.70) |  |  | Ref | 1.12 (0.63;2.02) | 1.66 (0.89;3.07) |  |  | Ref | 1.40 (0.37;5.23) | 0.88 (0.20;3.75) |  |
| 25-29.9 | Ref | | 0.61  (-0.92;2.14) | -1.14  (-2.78;0.51) |  |  | Ref | 1.15 (0.77;1.73) | 1.14 (0.74;1.75) |  |  | Ref | 1.07 (0.75;1.53) | 1.27 (0.85;1.88) |  |  | Ref | 1.33 (0.61;2.91) | 2.03 (1.00;4.11) |  |
| ≥30 | Ref | | -0.79  (-2.85;1.26) | -2.55  (-4.40;-0.71) | 0.71 |  | Ref | 0.95 (0.54;1.68) | 1.38 (0.83;2.30) | 0.65 |  | Ref | 0.93 (0.54;1.60) | 1.45 (0.85;2.49) | 0.92 |  | Ref | 1.06 (0.50;2.23) | 1.40 (0.76;2.59) | 0.80 |
| **Diabetes** |  | |  |  |  |  |  |  |  |  |  |  |  |  |  |  |  |  |  |  |
| No | Ref | | -0.19  (-1.39;1.00) | -1.72  (-2.98;-0.46) |  |  | Ref | 0.70 (0.30;1.63) | 1.23 (0.61;2.47) |  |  | Ref | 0.59 (0.28;1.25) | 1.29 (0.65;2.55) |  |  | Ref | 1.39 (0.54;3.58) | 0.98 (0.39;2.41) |  |
| Yes | Ref | | 1.44  (-1.51;4.38) | -1.22  (-3.89;1.45) | 0.60 |  | Ref | 1.05 (0.76;1.44) | 1.24 (0.89;1.73) | 0.65 |  | Ref | 1.14 (0.86;1.51) | 1.40 (1.02;1.91) | 0.26 |  | Ref | 1.11 (0.61;2.01) | 1.81 (1.09;3.00) | 0.29 |
| **Physical activity** |  | |  |  |  |  |  |  |  |  |  |  |  |  |  |  |  |  |  |  |
| Inactive | Ref | | -0.06  (-1.31;1.20) | -1.92  (-3.18;-0.67) |  |  | Ref | 1.10 (0.78;1.53) | 1.21 (0.86;1.69) |  |  | Ref | 1.00 (0.74;1.35) | 1.23 (0.89;1.68) |  |  | Ref | 1.21 (0.71;2.07) | 1.62 (1.01;2.58) |  |
| Active | Ref | | 0.32  (-2.04;2.68) | -0.52  (-3.09;2.06) | 0.62 |  | Ref | 0.70 (0.36;1.33) | 1.46 (0.76;2.81) | 0.32 |  | Ref | 1.22 (0.69;2.13) | 2.35 (1.25;4.42) | 0.19 |  | Ref | 1.09 (0.28;4.19) | 1.13 (0.29;4.33) | 0.88 |

ENRICA, Study on Nutrition and Cardiovascular Risk Factors in Spain.

OR: Odds ratio; CI: Confidence interval.

*p-value for the full model against the model with an interaction term

Beta coefficients and their 95% confidence intervals were obtained from multiple lineal regression models. Odds ratios and their 95% confidence intervals were obtained from multiple logistic regression models.

**Models** were adjusted for age, sex, educational level, body mass index (<25, 25-29.9, ≥30 kg/m2), tobacco (never-, ex-, current-smoker), total energy intake (kcal/day), MEDAS index, physical activity, cancer, diabetes, cardiovascular disease, osteomuscular disease and chronic respiratory disease. All linear regression models were also adjusted for the baseline PCS score.
