# Supplementary material for: Television viewing time as a risk factor for frailty and functional limitations in older adults: results from 2 European prospective cohorts
Source: Int J Behav Nutr Phys Act. 2017 Apr 26;14:54. doi: 10.1186/s12966-017-0511-1 (PMC5406978; doi:10.1186/s12966-017-0511-1)
Supplement: Supplementary file 2 — Stratified results for the association between tertiles of TV viewing time and incident limitations in physical function in older adults from the ELSA cohort. (DOCX 17 kb) [file 12966_2017_511_MOESM2_ESM.docx]

**Additional file 2: Table S2: Stratified results for the association between tertiles of TV viewing time and incident limitations in physical function in older adults from the ELSA cohort**

|  |  | **Mobility limitations**  OR (95% CI) | | | |  | **Agility limitations**  OR (95% CI) | | | |  | **Frailty**  OR (95% CI) | | | |
| --- | --- | --- | --- | --- | --- | --- | --- | --- | --- | --- | --- | --- | --- | --- | --- |
|  |  | **Tertiles of TV viewing time (h/day)** | | |  |  | **Tertiles of TV viewing time (h/day)** | | |  |  | **Tertiles of TV viewing time (h/day)** | | |  |
|  |  | **T1** ≤3 (M) or ≤3.6 (W) | **T2** 3-5 (M) or 3.7-5.6 (W) | **T3** >5 (M) or >5.6 (W) |  |  | **T1** ≤3 (M) or ≤3.6 (W) | **T2** 3-5 (M) or 3.7-5.6 (W) | **T3** >5 (M) or >5.6 (W) |  |  | **T1** ≤3 (M) or ≤3.6 (W) | **T2** 3-5 (M) or 3.7-5.6 (W) | **T3** >5 (M) or >5.6 (W) |  |
|  |  |  |  |  | **p*** |  |  |  |  | **p*** |  |  |  |  | **p*** |
| **Sex** |  |  |  |  |  |  |  |  |  |  |  |  |  |  |  |
| Men |  | Ref. | 1.05 (0.76;1.46) | 1.06 (0.74;1.52) |  |  | Ref. | 1.18 (0.86;1.61) | 1.03 (0.73;1.44) |  |  | Ref. | 1.20 (0.42;3.38) | 2.04 (0.82;5.11) |  |
| Women |  | Ref. | 0.97 (0.71;1.31) | 1.20 (0.87;1.66) | 0.70 |  | Ref. | 1.40 (1.03;1.88) | 1.29 (0.94;1.77) | 0.58 |  | Ref. | 1.00 (0.64;1.56) | 1.26 (0.82;1.93) | 0.59 |
| **BMI(kg/m^2^)** |  |  |  |  |  |  |  |  |  |  |  |  |  |  |  |
| <25 |  | Ref. | 1.01 (0.63;1.64) | 0.98 (0.57;1.68) |  |  | Ref. | 1.32 (0.86;2.00) | 0.75 (0.46;1.23) |  |  | Ref. | 0.65 (0.30;1.39) | 1.14 (0.58;2.24) |  |
| 25-29.9 |  | Ref. | 0.92 (0.65;1.30) | 1.16 (0.80;1.67) |  |  | Ref. | 1.32 (0.94;1.85) | 1.42 (0.99;2.04) |  |  | Ref. | 1.71 (0.84;3.47) | 2.17 (1.09;4.33) |  |
| ≥30 |  | Ref. | 1.13 (0.77;1.63) | 1.21 (0.82;1.79) | 0.89 |  | Ref. | 1.24 (0.85;1.81) | 1.18 (0.80;1.74) | 0.25 |  | Ref. | 0.91 (0.45;1.82) | 1.10 (0.57;2.12) | 0.36 |
| **Diabetes** |  |  |  |  |  |  |  |  |  |  |  |  |  |  |  |
| No |  | Ref. | 1.02 (0.81;1.30) | 1.16 (0.90;1.50) |  |  | Ref. | 0.59 (0.28;1.25) | 1.29 (0.65;2.55) |  |  | Ref. | 1.06 (0.69;1.62) | 1.28 (0.84;1.95) |  |
| Yes |  | Ref. | 0.83 (0.38;1.79) | 0.93 (0.44;1.97) | 0.83 |  | Ref. | 1.14 (0.86;1.51) | 1.40 (1.02;1.91) | 0.26 |  | Ref. | 10.94 (0.24;3.62) | 2.08 (0.63;6.90) | 0.46 |
| **Physical activity score** |  |  |  |  |  |  |  |  |  |  |  |  |  |  |  |
| Q1: <10 METs-h/week |  | Ref. | 1.02 (0.65;1.61) | 1.07 (0.67;1.72) |  |  | Ref. | 1.27 (0.84;1.92) | 1.23 (0.82;1.86) |  |  | Ref. | 0.84 (0.49;1.45) | 1.06 (0.63;1.78) |  |
| Q2: 10-16 METs-h/week |  | Ref. | 0.85 (0.61;1.20) | 1.32 (0.94;1.86) |  |  | Ref. | 1.28 (0.91;1.80) | 1.24 (0.86:1.79) |  |  | Ref. | 1.30 (0.67;2.53) | 1.41 (0.74;2.68) |  |
| Q3: >16 METs-h/week |  | Ref. | 1.26 (0.85;1.85) | 0.82 (0.50;1.33) | 0.06 |  | Ref. | 1.32 (0.90;1.93) | 0.96 (0.61;1.49) | 0.96 |  | Ref. | 1.04 (0.23;4.73) | 4.89 (1.46;16.4) | 0.11 |

ELSA: English Longitudinal Study of Ageing.

T: Tertile (T1, T2 and T3: Tertiles 1, 2 and 3); M: Men; F: Women. OR: Odds ratio; CI: Confidence interval.

*p-value for the full model against the model with an interaction term

Odds ratios and their 95% confidence intervals were obtained from multiple logistic regression models.

**Models** were adjusted for age, sex, educational level, body mass index (<25, 25-29.9, ≥30 kg/m^2^), tobacco (never-, former, current-smoker), physical activity, cancer, diabetes, cardiovascular disease, osteomuscular disease and chronic respiratory disease.
